# Extracellular Vesicles: The Invisible Heroes and Villains of COVID‐19 Central Neuropathology

**DOI:** 10.1002/advs.202305554

**Published:** 2023-12-24

**Authors:** Haiqing Chang, Erya Chen, Yi Hu, Lining Wu, Liyun Deng, Shixin Ye‐Lehmann, Xiaobo Mao, Tao Zhu, Jin Liu, Chan Chen

**Affiliations:** ^1^ Department of Anesthesiology West China Hospital Sichuan University Laboratory of Anesthesia and Critical Care Medicine National‐Local Joint Engineering Research Centre of Translational Medicine of Anesthesiology West China Hospital Sichuan University Chengdu Sichuan 610041 China; ^2^ Department of Cardiology, Honghui hospital Xi'an Jiaotong University Xi'an 710049 China; ^3^ Diseases and Hormones of the Nervous System University of Paris‐Scalay Bicêtre Hosptial Bât Grégory Pincus 80 Rue du Gal Leclerc, Cedex Le Kremlin Bicêtre 94276 France; ^4^ Department of Neurology Institute of Cell Engineering School of Medicine Johns Hopkins University Baltimore MD 21218 USA

**Keywords:** COVID‐19, central nervous system, extracellular vesicles, neuropathology, SARS‐CoV‐2

## Abstract

Acknowledging the neurological symptoms of COVID‐19 and the long‐lasting neurological damage even after the epidemic ends are common, necessitating ongoing vigilance. Initial investigations suggest that extracellular vesicles (EVs), which assist in the evasion of the host's immune response and achieve immune evasion in SARS‐CoV‐2 systemic spreading, contribute to the virus's attack on the central nervous system (CNS). The pro‐inflammatory, pro‐coagulant, and immunomodulatory properties of EVs contents may directly drive neuroinflammation and cerebral thrombosis in COVID‐19. Additionally, EVs have attracted attention as potential candidates for targeted therapy in COVID‐19 due to their innate homing properties, low immunogenicity, and ability to cross the blood‐brain barrier (BBB) freely. Mesenchymal stromal/stem cell (MSCs) secreted EVs are widely applied and evaluated in patients with COVID‐19 for their therapeutic effect, considering the limited antiviral treatment. This review summarizes the involvement of EVs in COVID‐19 neuropathology as carriers of SARS‐CoV‐2 or other pathogenic contents, as predictors of COVID‐19 neuropathology by transporting brain‐derived substances, and as therapeutic agents by delivering biotherapeutic substances or drugs. Understanding the diverse roles of EVs in the neuropathological aspects of COVID‐19 provides a comprehensive framework for developing, treating, and preventing central neuropathology and the severe consequences associated with the disease.

## Graphical abstract

On the one hand, EVs aid SARS‐CoV‐2 invasion in the nervous system. And the pro‐inflammatory, pro‐coagulant contents of EVs may drive lead to neuroinflammation and cerebral thrombosis in COVID‐19 neurological complications. On the other hand, EVs are potential candidates for prevention and treatment of COVID‐19 central neuropathology due to their homing properties, low immunogenicity, and ability to freely cross the blood‐brain barrier.

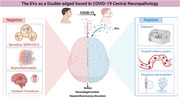

## 1 Introduction

The COVID‐19 pandemic, caused by the severe acute respiratory syndrome coronavirus 2 (SARS‐CoV‐2), has had a significant global impact since its emergence in late 2019.^[^ ^1^ ^]^ As of September 3, 2022, over 613 million reported infections and over 6.4 million deaths worldwide (https://covid19.who.int). While the initial focus of SARS‐CoV‐2 was on its respiratory effects,^[^ ^2^ ^]^ growing evidence indicates its involvement in a range of neurological diseases of varying severity.^[^ ^3^ ^]^ Neurological complications such as dizziness, headache, consciousness disturbance, hyposmia, hypogeusia, visual impairments, cerebrovascular disease (CVD), muscle pain, ataxia, and seizures have been observed in a large proportion of COVID‐19 patients.^[^ ^4^ ^]^ These complications may manifest during the onset of COVID‐19 (42.2%),^[^ ^5^ ^]^ at hospitalization (62.7%), or even as the initial symptoms (82.3%).^[^ ^6^, ^7^ ^]^ Some researchers suggest that the invasion of SARS‐CoV‐2 into the nervous system may contribute to fatal outcomes.^[^ ^8^ ^]^ However, the exact mechanism remains incomplete, and the relationship between SARS‐CoV‐2 and neurological deterioration has not yet been established.^[^ ^9^ ^]^

Extracellular vesicles (EVs), a cell‐to‐cell communication system with multifunctional roles in the human body,^[^ ^10^ ^]^ play a critical role in intercellular and interorgan communication across all domains of life.^[^ ^11^ ^]^ This vesicular transport pathway is critically involved in various aspects of human health and disease, including development, immunity, tissue homeostasis, cancer, and neurodegenerative diseases.^[^ ^12^ ^]^ EVs are recognized as potential information carriers implicated in neuropathological processes, leading to neurocognitive dysfunction and brain damage by crossing the BBB or neural pathways.^[^ ^13^ ^]^ Substantial evidence suggests that the neuropathological characteristics of COVID‐19 arise primarily from two mechanisms: direct virus invasion, via a “Trojan horse” mechanism whereby the virus enters the brain through neural pathways or circulation^[^ ^14^ ^]^ and systemic deteriorations induced by respiratory diseases, such as inflammation and coagulation dysfunction.^[^ ^9^, ^15^ ^]^ EVs have been implicated in the pathogenesis of COVID‐19, participating in viral infections, inflammatory storms, thrombosis, and more.^[^ ^16^ ^]^ Additionally, EVs’ involvement in virus‐related cognitive dysfunctions, such as HIV‐associated neurological disorders (HANDs), is well‐established.^[^ ^17^ ^]^ Yet, the exact role of EVs in neurological complications arising from SARS‐CoV‐2 infection remains incompletely understood. In light of this, we hypothesize that EVs partially mediate the neurological complications observed during SARS‐CoV‐2 infection.

Moreover, a growing body of research focuses on the potential therapeutic applications of EVs from mesenchymal stromal cells (MSCs) and other cellular sources in treating COVID‐19.^[^ ^18^ ^]^ However, little attention has been paid to the potential therapeutic role of EVs in COVID‐19‐associated neurological complications. In this review, by describing COVID‐19‐related neurological symptoms and complications, we investigate the mechanisms of EV in SARS infection and subsequent neuropathological changes. Furthermore, we discuss the diagnostic and therapeutic roles of EVs in COVID‐19‐related neurological disorders.

## 2 Neurological Symptoms, Manifestation, and Complications of COVID‐19

### 2.1 Neurological Symptoms and Complications of COVID‐19

**Figure 1:**
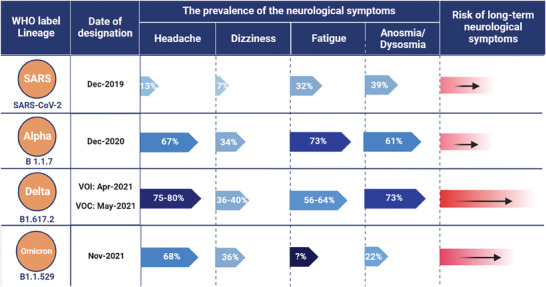
Strain variation and neurological symptoms of SARS‐CoV‐2. The incidence of neurological symptoms caused by SARS‐CoV‐2 gradually increased as the strain mutates, peaking in delta and showing a mildly decreasing trend in omicron (where the incidence of peripheral nervous system symptoms of olfactory disturbance or loss of smell decreased substantially). Furthermore, although the exact incidence of long‐term neurological symptoms caused by SARS‐CoV‐2 is not available, 6‐month (omicron: over 140 days of follow‐up) risks of neurological symptoms increase with the evolution of the strain. VOI, variant of interest; VOC: variants of concern.

Increasing evidence has substantiated the existence of neurological complications linked to both COVID‐19 infection and vaccinations. The incidence of neurological symptoms caused by SARS‐CoV‐2 also increased as strains mutated^[^ ^19^ ^]^ (**Figure** **1**). In the recent wave of outbreaks, headache emerged as the second most common symptom among participants who tested positive for the delta variant (77.9%) or omicron variant (74.7%), following a runny nose.^[^ ^19a^ ^]^ In addition to headache, the central nervous system (CNS) symptoms include dizziness, lethargy, unstable gait, impaired consciousness, ataxia, and seizure. Peripheral nervous system (PNS) symptoms consist of taste impairment, smell impairment, vision impairment, nerve pain, and malaise.^[^ ^3^, ^20^ ^]^ Notably, CNS symptoms were the predominant manifestations in patients with COVID‐19‐related neurological abnormalities.^[^ ^20a^ ^]^ The neurological symptoms associated with COVID‐19 varied greatly depending on the age group. Seizures or status epilepticus are more likely to occur in younger patients, while anosmia and/or ageusia, headache, and fatigue/weakness are more common in older patients.^[^ ^21^ ^]^ A substantial body of evidence indicates that SARS‐CoV‐2 is likely to persist long‐term and leave a significant number of survivors with unexpected long‐term neurological consequences, such as joint pain, chest pain, sleep difficulties, cognitive impairment (referred to as “brain fog”), dementia, epilepsy, and seizures.^[^ ^19^, ^22^ ^]^ Cognitive impairment refers to one or more deficits in memory, language, visuospatial, execution, calculation, comprehension, and judgment. The risk of long‐term cognitive decline is increased in the elderly population with SARS‐CoV‐2 infection.^[^ ^23^ ^]^ A large cohort study conducted in China demonstrated that the incidence of cognitive impairment in survivors 12 months after discharge was 12.45%.^[^ ^24^ ^]^ Furthermore, the neurological involvement of COVID‐19 encompasses complications in CNS and PNS.

#### 2.1.1 CNS Inflammatory Disorders

Recent studies have indicated that the coronavirus can attack the brain through multiple mechanisms.^[^ ^4^ ^]^ Initially, meningitis was reported as a result of SARS‐CoV‐2 infection.^[^ ^5^ ^]^ CNS inflammatory disorders, including myelitis, acute disseminated encephalomyelitis, acute hemorrhagic necrotizing encephalitis, and cytotoxic lesion of the corpus callosum, are more prevalent in cases of severe COVID‐19 infection that result in fatalities.^[^ ^25^ ^]^

#### 2.1.2 Stroke

CVD are most frequently diagnosed neurological consequence of COVID‐19, however, patients with CVD (76%) experience adverse outcomes more frequently than those with encephalopathy (54%).^[^ ^26^ ^]^ In a comprehensive nationwide study conducted in the UK, it was observed that roughly 62% of COVID‐19 patients monitored through an online portal displayed cerebrovascular sequelae, such as ischemic stroke, mental status changes, and encephalopathy.^[^ ^1^ ^]^ According to The American Heart Association/American Stroke Association, roughly 6% of COVID‐19 patients will encounter a stroke within 10 days of disease onset.^[^ ^27^ ^]^ Meta‐analysis reveals that the combined incidence of acute ischemic stroke is ≈1.2%, with ≈40% resulting in mortality.^[^ ^28^ ^]^ These figures, along with the occurrence of stroke in young patients devoid of cerebrovascular disease risk factors or identifiable sources of thrombosis, offer limited evidence supporting the pro‐coagulopathy state induced by COVID‐19.

#### 2.1.3 Neurodegeneration

Although CVD and encephalopathy are more commonly observed in COVID‐19 and tend to occur in the early stages of the pandemic,^[^ ^29^ ^]^ it is crucial to emphasize that the neurological damage caused by SARS‐CoV‐2 extends far beyond these short‐term neuropathological manifestations. COVID‐19‐associated symptoms encompass cognitive, motor, and non‐motor manifestations, including Guillain‐Barré syndrome, polyneuropathy, and parkinsonism.^[^ ^30^ ^]^ These symptoms suggest the occurrence of degenerative and demyelinating processes within the CNS, alongside systemic inflammation and ischemia. Notably, COVID‐19 often leads to heightened ferritin levels, which may contribute to a mechanism known as ferrosenescence. Ferrosenescence entails viral replication in cells with abundant iron, potentially disrupting immune regulation and resulting in subsequent neurodegeneration in COVID‐19.^[^ ^31^ ^]^ Furthermore, the virus can directly cause neurodegeneration through its neurotropic capabilities.^[^ ^32^ ^]^

#### 2.1.4 Spinal Cord Injury

Rare cases of neuromyelitis optica, characterized by an autoimmune attack on the spinal cord and optic nerves, as well as instances of paraplegia resulting from an epidural abscess and cervicothoracic myelopathy in COVID‐19 patients, have been reported in the literature.^[^ ^33^ ^]^ These reports confirm that COVID‐19 indeed has an impact on the spinal cord. The process of spinal cord injury follows a similar sequence of events as seen in stroke, involving neuroinflammation, damage, and death of various types of glial cells (including neurons, microglia, astrocytes, and oligodendrocytes), formation of cavities, disruption of spinal cord circuitry, and altered neuronal activity.

Remarkably, COVID‐19 can lead to respiratory failure, which is considered a pulmonary symptom. However, it is worth noting that this respiratory failure may be partly due to the involvement of the brainstem by SARS‐CoV2.^[^ ^34^ ^]^ As proposed by Song et al, SARS‐CoV‐2 can potentially infect the sensory nerves responsible for regulating cough in post‐COVID syndrome, which may involve neuroimmune factors.^[^ ^35^ ^]^ In essence, the pulmonary clinical manifestations observed in the ongoing pandemic may have underlying neurological implications.

### 2.2 Effects of COVID‐19 on Central Neurological Diseases

COVID‐19 has the potential to exacerbate manifestations of pre‐existing neurological diseases, especially neurodegenerative diseases.^[^ ^36^ ^]^ Parkinson's disease (PD), characterized by four primary motor symptoms (tremor, rigidity, akinesia, and postural instability), also presented with prominent non‐motor symptoms.^[^ ^37^ ^]^ A substantial amount of evidence revealed that PD patients are more susceptible to SARS‐CoV‐2, and SARS‐CoV‐2 contributed to worse motor and nonmotor performance and potentially increased mortality in patients with advanced stages of the disease.^[^ ^38^ ^]^ Similarly, Brown et al. reported new or worsening motor (63%) and non‐motor (75%) symptoms in PD patients diagnosed with or testing positive for COVID‐19, with women being at higher risk.^[^ ^39^ ^]^ Alzheimer's disease (AD) is characterized by cognitive decline and non‐cognitive psychiatric symptoms as the disease progresses. Prolonged lockdowns and confinement during the COVID‐19 pandemic have been linked to cognitive deterioration in AD patients,^[^ ^40^ ^]^ and SARS‐CoV‐2 infection exacerbates AD status by increasing neurotoxicity.^[^ ^41^ ^]^ AD patients show vulnerability to COVID‐19,^[^ ^42^ ^]^ and in turn, COVID‐19 contributes to the occurrence of AD.^[^ ^41^, ^43^ ^]^ This mutually reinforcing cycle poses a significant challenge to AD patients during the COVID‐19 pandemic,^[^ ^44^ ^]^ with numerous studies demonstrating a significantly increased mortality rate in AD patients following SARS‐CoV‐2 infection.^[^ ^45^ ^]^ Moreover, evidence suggests that SARS‐CoV‐2 may accelerate neurodegeneration, as seen in Creutzfeldt‐Jakob disease (CJD), a chronic, progressive, and fatal CNS disorder.^[^ ^46^ ^]^ Patients with CJD display progressive neurological symptoms, such as severe declines in cognition, memory, and executive functioning after SARS‐CoV‐2 infection.^[^ ^47^ ^]^ There are also studies linking SARS‐CoV‐2 to the development or progression of another neurodegenerative cognitive disorder, frontotemporal dementia (FTD).^[^ ^48^ ^]^ Apart from neurodegenerative diseases, COVID‐19 is associated with deterioration of cerebrovascular complications. However, COVID‐19 patients with a history of stroke exhibit a higher incidence of CVD, more comorbidities, poorer outcomes, and higher mortality than COVID‐19 patients without a history of stroke.^[^ ^49^ ^]^ Subtype analysis showed that SARS‐CoV‐2 infection leads to an increased risk of hemorrhagic stroke and ischaemic attack (TIA) in patients with a history of ischaemic stroke and augments the risk of hemorrhagic stroke in patients with a history of hemorrhagic stroke.^[^ ^50^ ^]^ This implies that SARS‐CoV‐2 not only exacerbates pre‐existing CVD subtypes but also serves as a potential trigger for other types of cerebrovascular events. These findings suggest that SARS‐CoV‐2, as a facilitator of neurological disease, may worsen already severe neurological deficits. However, the modulation of neuropathology progression by SARS‐CoV‐2 outside the CNS has yet to be elucidated.

## 3 EVs and SARS‐CoV‐2 Infection

### 3.1 EVs

EVs refers to a heterogeneous group of membranous vesicles, including exosomes, microvesicles, microparticles, tumor bodies, and prostatic bodies, with sizes ranging from 20 to 500 nm.^[^ ^9^, ^51^ ^]^ These membranous organelles can be released by cells from all three domains of life—Archaea, Bacteria, and Eukarya.^[^ ^52^ ^]^ In addition, EVs contain a diverse array of molecules, such as proteins, lipids, DNA, mRNAs, microRNAs (miRNAs), circular RNAs (circRNAs), long non‐coding RNAs (lncRNAs), and pathogen‐derived nucleic acids.^[^ ^53^ ^]^ They can be detected in various body fluids, including plasma, ascites, saliva, breast milk, urine, ejaculate, amniotic fluid, and cerebrospinal fluid (CSF).^[^ ^54^ ^]^ EVs play pivotal roles in intercellular and interorgan communication, as well as in lateral or horizontal gene transfer. Furthermore, they participate in responses to stimuli and infectious agents, such as bacteria, fungi, parasites, and viruses, by transporting their molecular cargo.^[^ ^55^ ^]^

### 3.2 EVs and Virus

**Figure 2:**
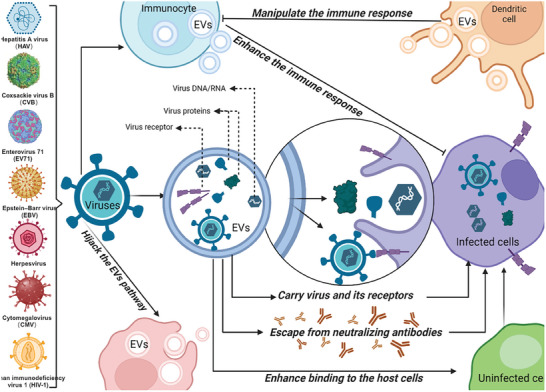
EVs and Viruse. Numerous viruses, such as HAV, CVB, EV71, EBV, herpesvirus CMV and HIV‐1, use EVs as vectors to enter host cells, facilitate viral transmission, evade immune responses, and expand their territories in the following ways: Using EVs to carry intact viruses or viral components and viral receptors; using EVs membrane envelopes to evade recognition by neutralizing antibodies; using EVs surface proteins to enhance their tropism in the host; using EVs to evade antiviral responses; using EVs to manipulate and control immune responses; holding EVs pathways hostage to interfere with EVs function. Conversely, viruses can also stimulate immune cells to release EVs carrying antigenic peptides, which enhance the immune response and are one of the protective mechanisms of the organism. HAV: Hepatitis A virus; CVB: Coxsackie virus B; EV71; Enterovirus 71; EBV: Epstein–Barr virus; CMV: Cytomegalovirus; HIV‐1: Human immunodeficiency virus 1.

Viruses are small, non‐cellular organisms with a simple structure consisting of only one nucleic acid (DNA or RNA). Interestingly, viruses, particularly enveloped viruses, share numerous striking similarities with EVs. These parallels are evident in their physicochemical properties (similar density, size, and heterogeneous size distribution) and mechanisms for biogenesis and cell entry.^[^ ^56^ ^]^ Even more surprisingly, viruses exploit EVs as vectors to enter host cells, facilitate viral spread, evade immune responses, and expand their territories.^[^ ^56^, ^57^ ^]^ On the one hand, EVs are susceptible to viral exploitation. Compelling evidence shows that EVs isolated from virus‐infected cells contain intact viruses,^[^ ^58^ ^]^ as well as various viral components such as proteins, genomic RNAs, and miRNAs.^[^ ^59^ ^]^ While the spread of viruses was traditionally thought to occur through individual free virus particles, it is now increasingly recognized that viruses can also employ EVs for vesicle‐mediated en bloc transmission.^[^ ^60^ ^]^ These EV‐encapsulated viral clusters, whether enveloped or non‐enveloped, are considered distinct forms of the infectious unit.^[^ ^61^ ^]^ Certain non‐enveloped picornaviruses, including hepatitis A virus, Coxsackie virus B, and Enterovirus 71 (EV71), have been observed to be released within EVs, thus acquiring a “cellular envelope” of sorts.^[^ ^62^ ^]^ It is hypothesized that enveloped viruses within EVs may exploit this membrane coating to evade recognition by neutralizing antibodies, and enhance their tropism within the host by utilizing cellular surface proteins.^[^ ^63^ ^]^ On the other hand, viruses induce immune cells to produce EVs and manipulate immune responses.^[^ ^64^ ^]^ Epstein–Barr virus (EBV) is a notable example of a DNA virus that utilizes vesicular production to evade the antiviral response. EBV‐infected cells release vesicles that contain an abundance of viral proteins to induce immune modulation^[^ ^65^ ^]^ and upregulate the expression of adhesion molecules in uninfected cells, rendering them more susceptible to infection.^[^ ^66^ ^]^ Kaposi's sarcoma‐associated herpesvirus (KSHV/HHV‐8) instead controls the packaging of host factors. EVs released by KSHV‐infected cells carry an abundance of metabolic proteins and immune‐related proteins, resulting in alternations to recipient cells’ metabolism and immune response, ultimately facilitating viral persistence;^[^ ^67^ ^]^ similar process is observed in herpes simplex virus^[^ ^68^ ^]^ and cytomegalovirus (CMV). For instance, human endothelial cells infected with cytomegalovirus transferred their exosome‐like particles to antigen‐presenting cells, stimulating allogeneic CD4^+^ memory T cells.^[^ ^69^ ^]^ Immature dendritic cells (DCs) can capture human immunodeficiency virus‐1 (HIV‐1) particles and transmit them to T cells through exosomes.^[^ ^70^ ^]^ EVs from HIV‐infected cells have been found to contain their target cell receptors, C‐C chemokine receptor type 5 (CCR5) or recombinant chemokine C‐X‐C‐motif receptor 4 (CXCR4), which aid in infectivity.^[^ ^71^ ^]^ DC‐derived exosomes also play a role in activating naive CD4^+^ T cells and carrying antigen peptides, thereby enhancing the initiation of adaptive immune responses.^[^ ^72^ ^]^ Besides, viruses can interfere with EV's function and hijack EVs pathways to ensure their survival and persistence.^[^ ^63^, ^73^ ^]^ Infected cells released exosomes with different protein profiles and amounts compared to uninfected cells, which is associated with cell death and survival, as seen in HIV‐1, Epstein‐Barr virus (EBV), and Kaposi sarcoma‐associated virus (KSHV).^[^ ^74^ ^]^ In summary, the involvement of EVs in viral infection extends beyond assisting virus assembly,^[^ ^75^ ^]^ encompassing features such as expanded cell and tissue tropism of the virus.^[^ ^76^ ^]^ These characteristics make viruses released from EVs more infectious than free viruses and enable them to infect cells that would otherwise be resistant to infection (**Figure** **2**).

### 3.3 EVs and SARS‐CoV‐2

The potential role of EVs in SARS‐CoV‐2 infection is an emerging and dynamic research area. Previous studies have demonstrated that EVs contribute to the progression of COVID‐19. Notably, plasma EVs from patients with COVID‐19 pneumonia contained 43 significantly differentially expressed miRNAs compared to healthy donors.^[^ ^77^ ^]^ Furthermore, circulating EVs in SARS‐CoV‐2‐positive patients exhibited distinct proinflammatory, procoagulant, and tissue remodeling features, distinguishing them from uninfected controls. These plasma EVs from hospitalized COVID‐19 patients can differentiate between moderate and critically ill patients and have also been shown to promote apoptosis of pulmonary vascular endothelial cells.^[^ ^78^ ^]^ Interestingly, Kudryavtsev et al. found that the concentration of certain EVs correlated with the severity of lung injury in COVID‐19 patients. While plasma CD235a^+^ and CD14^+^ EVs levels were significantly elevated in moderately infected patients compared to healthy donors, CD8^+^ and CD19^+^ EVs levels decreased.^[^ ^79^ ^]^ More direct evidence is that virions or their components can accumulate in EVs. For example, the SARS‐CoV‐2 spike protein was found to be incorporated into EVs of HEK293T cells.^[^ ^80^ ^]^ The presence of SARS‐CoV‐2 spike protein 1 (S1) and its receptor‐binding domain (RBD) in exosomes from the plasma of COVID‐19 patients further confirmed this observation.^[^ ^81^ ^]^ Furthermore, SARS‐CoV‐2 RNA has been detected in EVs, suggesting possible transmission of the virus via EVs.^[^ ^82^ ^]^ Recent findings by Eymieux et al. using transmission electron microscopy demonstrated that intracellular SARS‐CoV‐2 transport occurs through small secretory vesicles, which release virus particles upon fusion with the plasma membrane.^[^ ^83^ ^]^ This suggests that secretory vesicles are the primary means by which SARS‐CoV‐2 is expelled from cells, which is plausible given the size of coronaviruses at ≈60–140 nm.^[^ ^84^ ^]^ Some scholars have even speculated that EVs may serve as a reservoir for SARS‐CoV‐2 in recovered COVID‐19 patients, potentially contributing to reinfection or reactivation after extended periods of recovery.^[^ ^85^ ^]^ Moreover, EVs offer a stable means of carrying SARS‐CoV‐2, as demonstrated in non‐human primate models, where SARS‐CoV‐2‐containing EVs were detected as early as day 1 post‐infection, plateauing from day 6 to day 28.^[^ ^86^ ^]^ Accordingly, the development of new technologies for the detection of SARS‐CoV‐2 exosomes has become necessary and has subsequently emerged.^[^ ^87^ ^]^ In conclusion, EVs represent one of the strategies employed by SARS‐CoV‐2 to evade immune responses, potentially facilitating the transfer of viral components and pathological signals to other cells or organs through the transfer of proteins, miRNAs, and other characteristic substances involved in the pathogenesis of COVID‐19 infection.

## 4 EVs in SARS‐CoV‐2‐Induced Neuropathology

The involvement of EVs in neuropathology has garnered significant attention due to their potential role in transferring pathological “seeds”, including amyloid‐beta (Aβ) and tau, polyglutamine (poly Q) protein, TAR DNA‐binding protein 43(TDP‐43) and α‐synuclein.^[^ ^88^ ^]^ Various types of EVs have been shown to bidirectionally cross the BBB in many pathophysiological processes.^[^ ^89^ ^]^ The carriage and transport characteristics make EVs provide a potential pathway for peripheral viruses to enter the brain, leading to alternation in glial activity, upregulation of neuroinflammation, and triggering of neurotoxicity.^[^ ^90^ ^]^ Furthermore, EVs have been identified as important sources of prion infectivity, carrying the transmissible spongiform encephalopathy‐associated prion protein (PrPTSE) and transmitting neurotoxicity.^[^ ^91^ ^]^ Additionally, EVs may serve as possible candidates for spreading PrPTSE from distal organs to the brain.^[^ ^92^ ^]^ Enteroviruses, such as Enterovirus A71 and Enterovirus D68, have been found to enter the CNS via the EVs pathway, leading to neuroinflammation and neuronal degeneration.^[^ ^93^ ^]^ HIV accessory protein negative factor (Nef) and other viral components can also be packaged into EVs, contributing to HANDs.^[^ ^94^ ^]^ Moreover, the exosome‐associated trans‐activated regulatory protein (Tat) induces neurotoxicity as evidenced by neuronal shortening and reduced neuronal survival.^[^ ^94b^ ^]^ In addition to delivering viruses or viral components to the CNS, EVs can also transport inflammatory mediators to promote neuropathology. For instance, elevated miR‐21 expression has been observed in EVs isolated from simian immunodeficiency virus encephalitis (SIVE) brains, and the delivery of miR‐21 in EVs‐like vesicles is critical for the TLR7‐dependent initiation of neurotoxicity.^[^ ^95^ ^]^ Hence, there is irrefutable evidence that EVs play a critical role in viral infiltration into the CNS and developing virus‐related neuropathology.

Recent research has confirmed that SARS‐CoV‐2 infection not only leads to certain neurological symptoms but also causes significant abnormalities in brain structure. A study conducted by Douaud et al. showed a smaller overall brain size in individuals with SARS‐CoV‐2 infection. Alongside cognitive decline, there were notable reductions in gray matter thickness and tissue contrast in the frontal cortex and parahippocampal gyrus.^[^ ^96^ ^]^ As our understanding of the neuropathology of COVID‐19 continues to expand, it is crucial to recognize and address the detrimental effects of this disease on the nervous system, particularly the CNS. Moreover, given the close association between EVs and COVID‐19‐related neuropathological manifestations (**Table** **1**), we aim to provide a comprehensive summary of the role of EVs in COVID‐19‐related neuropathology.

**Table 1:** Involvement and predictive role of EVs in COVID‐19 neurological manifestations and neuropathology.

EVs Sources	Cargo	Results	Ref
Plasma (neuron)	Aβ neurofilament light neurogranin total tau p‐T181‐tau	Persistent peripheral and neurological inflammation following COVID‐19 infection may affect neurological sequelae by altering neuronal EV proteins. These neuronal EV proteins may be a potential plasma biomarker for ongoing neuroinflammation or neurodegeneration	[97]
Plasma (neuron, astrocyte)	SARS‐CoV‐2 N and S1 protein mitochondrial proteins (MPs)	Abnormal NDEV and ADEV levels of SARS‐CoV‐2 N and S1 protein and MPs correlate with neuropsychiatric manifestations and may be biomarkers for long‐COVID prognostics and therapeutic trials	[98]
Plasma (endothelial cells)	miR‐24	Plasma endothelial EV miR‐24 levels are related to cerebrovascular events in COVID‐19 hospitalized patients	[99]
Lung	B‐cell CLL/lymphoma 3 (BCL3) Jun D proto‐oncogene (JUND) MAX dimerization protein 1 (MXD1) Interferon regulatory factor 2/9 (IRF2/9) Signal transducer and activator of transcription 1 (STAT1)	In SARS‐CoV‐2 infection, BCL3, JUND, MXD1, IRF2, IRF9, and STAT1 transcription factors in EVs affect neuronal gene regulatory networks and accelerate neurodegeneration	[100]
Plasma	mitochondrial open‐reading‐frame of the twelve S rRNA‐c(MOTS‐c) humanin, Sterile Alpha and TIR Motif‐Containing Protein 1(SARM‐1)	Significant decreases in total extracellular vesicle levels of MOTS‐c and humanin, and elevations of total extracellular vesicle levels of SARM‐1 were characteristic of post‐acute sequelae of COVID‐19 patients with neuropsychiatric manifestations.	[101]
plasma cerebrospinal fluid	complement proteins	Patients with COVID‐19 with neurological manifestations have EVs in their cerebrospinal fluid and higher expression of complement proteins on circulating plasma EVs	[102]
Lung	Nucleoporin 62 (p62) Ras‐related protein Rab‐7A (RAB7A) Ras GTPase‐activating protein‐binding protein 1 (G3BP1) Microtubule affinity regulating kinase 2 (MARK2) Osteosarcoma amplified 9 (OS9), etc.	Interfering with the autophagic/ubiquitination process triggers the production of large amounts of EVs containing perturbants that damage several proteins susceptible to PD, which may trigger Parkinson's disease in COVID‐19 patients	[103]

### 4.1 Invasion of the CNS by SARS‐CoV‐2

The presence of neurological symptoms has prompted the investigation into the potential neurotropism of SARS‐CoV‐2, i.e., its ability to infect and replicate in nervous system cells.^[^ ^104^ ^]^ Current studies indicate that SARS‐CoV‐2 primarily targets choroid plexus epithelial cells, and may also attack neurons and astrocytes,^[^ ^105^ ^]^ although replication in nerve cells is still uncertain.^[^ ^105^, ^106^ ^]^ In experiments with non‐human primates, viral RNA was discovered in the CSF, olfactory triangle, and the entorhinal region, with the viral antigen nucleoprotein (NP) gradually spreading throughout the brain.^[^ ^107^ ^]^ It is suggested that direct infection with SARS‐CoV‐2 contributes to neurological symptoms, but a significant portion of COVID‐19 neuropathology may be caused by non‐SARS‐CoV‐2 infections.^[^ ^108^ ^]^

### 4.2 EVs, SARS‐CoV‐2's Ride into the CNS

Exosomes originating from highly susceptible peripheral tissues, such as the intestine and lungs, could potentially reach the brain through retrograde axonal transport along peripheral nerves.^[^ ^109^ ^]^ According to Ahmed et al., exosomal transcriptional factors have the ability to regulate gene expression and induce neuronal changes associated with neurodegeneration.^[^ ^100^ ^]^ Among the 19 overexpressed exosomal transcriptional factors during the acute phase of SARS‐CoV‐2 infection, BCL3, JUND, MXD1, IRF2, IRF9, and STAT1 were observed to activate genes linked to PD in key brain regions, including the substantia nigra and superior frontal gyrus. These genes are involved in vital functions such as signal transduction, neuronal cell death, and immune surveillance, whose dysregulation could potentially contribute to neurodegeneration. For instance, STAT1 activation leads to microglial activation and autophagy of dopaminergic neurons in hypoxic conditions, as seen in COVID‐19‐induced hypoxia.^[^ ^110^ ^]^ However, a postmortem study on COVID‐19 patients revealed significant neurological damage; however, minimal levels of SARS‐CoV‐2 RNA were detected in their brains.^[^ ^111^ ^]^ Building upon the Braak hypothesis, it is plausible to suggest that the neurological consequences and neuropathological manifestations observed in COVID‐19 survivors may arise from both direct effects of the virus and indirect effects mediated by molecular and neuroinflammatory factors carried by SARS‐CoV‐2‐related EVs that persist in circulation even after viral elimination.

Angiotensin‐converting enzyme 2 (ACE2) is widely recognized as the primary receptor of SARS‐CoV‐2, facilitating its pathogenic effect.^[^ ^112^ ^]^ Consequently, areas of the brain where ACE2 is present may serve as docking points for SARS‐CoV‐2 following its entry into the brain.^[^ ^113^ ^]^ The single‐nucleus RNA sequencing of the olfactory bulb and prefrontal cortex highlighted a remarkable diversity of coronavirus receptors, with ACE2 rarely expressed.^[^ ^114^ ^]^ This appears to be consistent with the low amount of viral RNA in the frontal cortex of patients who died of COVID‐19.^[^ ^115^ ^]^ If SARS‐Cov2 directly invades the nervous system and causes disease, this implies that the area of distribution of SARS‐Cov2 in the brain after infection should also have the distribution of ACE2. However, analyzing publicly available brain transcriptome databases, it is apparent that ACE2 is generally low in the brain but is relatively highly expressed in certain brain regions, such as the choroid plexus and paraventricular nucleus of the thalamus.^[^ ^116^ ^]^ However, this contrasts with current understanding as limited human research supports SARS‐Cov2 presence in these regions with high ACE2 expression (e.g., choroid plexus). Conversely, SARS‐Cov2 has been detected in other brain area (olfactory bulb, amygdala, entorhinal area, temporal and frontal neocortex, dorsal medulla, and pia mate).^[^ ^117^ ^]^ This suggests a possible mechanism for the viral receptor ACE2 to be packaged into EVs.^[^ ^118^ ^]^ At the same time, the cells expressing ACE2 and CD9 may transfer these SARS‐CoV‐2 viral receptors to cells that do not express these receptors via EV, making other cells more susceptible to SARS‐CoV‐2 infection.^[^ ^16^ ^]^

**Figure 3:**
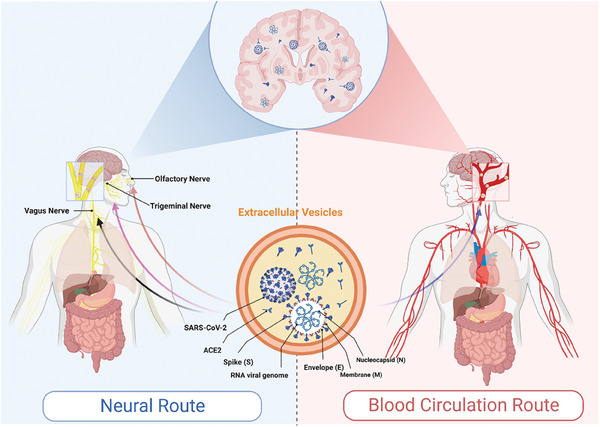
EVs assist SARS‐CoV‐2 in entering the CNS. Upon infection with SARS‐CoV‐2, the body releases large numbers of extracellular vesicles (EVs) containing SARS‐CoV‐2 and its components (e.g., RNA, nucleic acids, spike protein) and receptors (Angiotensin‐converting enzyme 2, ACE2). Then, SARS‐CoV‐2 facilitates its neural invasion through two pathways with the help of this cryptic EV transport. One is that EVs carrying SARS‐CoV‐2‐associated contents cross the blood‐brain barrier into the central nervous system (CNS) via circulation. In addition, EVs also can carry SARS‐CoV‐2‐associated contents retrogradely into the CNS via the vagus, trigeminal, or olfactory nerves.

It has been hypothesized that EVs may play a role in intercellular communication and contribute to the spread of SARS‐CoV‐2 from the lungs to other organs, especially establishing communication with the nervous system.^[^ ^100^ ^]^ Analysis of EVs from various human and murine cell lines revealed that all EVs were capable of crossing the mouse BBB, with four exosomes exhibiting a preference for uptake by the olfactory bulb.^[^ ^119^ ^]^ EVs possess an inherent ability to conceal viral cargoes from host immune surveillance, resulting in immune evasion and facilitating the spread of viruses in the bloodstream. A study has even suggested SARS‐CoV‐2 virions can enter cells without ACE2 receptor involvement when attached to microparticles.^[^ ^120^ ^]^ The infrequency of SARS‐CoV‐2 RNA found in the brain indicates that active viral infection and replication are likely not occurring within the brain.^[^ ^115^ ^]^ However, partial SARS‐CoV‐2 components or complete viral particles may be concealed within EV vesicles, utilizing a “Trojan horse” strategy for passive transport into the nervous system.^[^ ^121^ ^]^ Existing evidence suggests that SARS‐CoV‐2 enters the nervous system through two routes (**Figure** **3**). The first is via blood circulation, in which the virus gains access to the CNS through the BBB or the blood‐cerebrospinal fluid barrier. As previously mentioned, EVs derived from the plasma of COVID‐19 patients contain viral proteins or RNA components, and patients with COVID‐19 are at risk of BBB impairment.^[^ ^81^, ^82^, ^122^ ^]^ In fact, several studies have demonstrated that EVs have an inherent ability to traverse the BBB via transcellular migration, even without virus infections.^[^ ^123^ ^]^ Furthermore, impaired neurological barriers in a proinflammatory environment enhance EVs transmigration into the brain.^[^ ^124^ ^]^ Therefore, it is reasonable to assume that EVs have the capacity to transport SARS‐CoV‐2 from the periphery to the CNS through the circulation. The second route is the neural pathway, wherein the virus enters neural tissue via retrograde and anterograde transport along peripheral nerves. The major neural pathways involved are the olfactory, trigeminal, and vagus nerve.^[^ ^20^, ^125^ ^]^ Currently, the olfactory nerve pathway is the most recognized route for SARS‐CoV‐2 transmission.^[^ ^107^, ^126^ ^]^ Intranasal administration of EVs proves to be an effective means of delivering their contents to the brain and exerting their effects.^[^ ^127^ ^]^ Furthermore, the possibility of SARS‐CoV‐2 transmission through the vagus nerve fibers has been proposed.^[^ ^109^, ^128^ ^]^ EVs have also been shown to retrogradely enter the brain via the vagus nerve and induce neuropathology.^[^ ^13c^ ^]^

In addition to serving as carriers of the virus, SARS‐CoV‐2 infection results in significant changes in the composition of EVs, and further enhances the migration of EVs to the brain. Exosomal modification by a class of sialylated lipids called monosialodihexosyl gangliosides was found to display a positive association with the disease severity in plasma EVs isolated from patients with COVID‐19.^[^ ^129^ ^]^ Previous research has shown that increased sialylation on nanoparticles enhances their ability to permeate the BBB through electrostatic interactions with N‐acetylglucosamines on human brain microvascular endothelial cells, which make up the BBB.^[^ ^130^ ^]^ Additionally, the virus‐induced elevation of exosomal CD147 (a neuron‐glia interactions‐implicated cell recognition molecule) and gangliosides with sialic acids can promote exosome transmigration across the BBB, facilitating viral neurotropism.^[^ ^131^ ^]^ Virus‐driven neurological abnormalities, mediated by HDLs and exosomes, are influenced by lipid rafts, which play a role in the production and movement of these lipid particles across the blood‐brain barrier (BBB). Lipid rafts are not only vital to the natural formation and neurotropic behavior of both HDLs and exosomes, but they also have the ability to adjust the cargo composition and biological characteristics of exosomes, consequently affecting their neurological impact. Regarding neurotropism, it is worth noting that ACE2 and SR‐BI are found on lipid rafts. The composition of lipid rafts in exosomes determines the preferred location of membrane proteins associated with rafts, such as PS‐1. Elevated levels of lipid raft lipids result in increased membrane rigidity and higher placement of PS‐1 on plasma exosomes during the hyperinflammatory phase of COVID‐19. GS enzyme cleaves NOTCH‐1, leading to interleukin‐6 production in a cycle of positive feedback, sustaining systemic inflammation that drives the hyperinflammatory stage of COVID‐19. GS also cleaves APP, generating Aβ peptides linked to cognitive impairment. A small group of COVID‐19 patients showed higher levels of plasma Aβ40 peptides during the hyperinflammatory phase compared to the resolution phase. Therefore, the lipid raft composition influences the biological characteristics of exosomes and may enhance their movement across the BBB, impacting brain metabolism. Thus, during the hyperinflammatory phase of COVID‐19, exosomes carrying viral materials and proinflammatory proteins can more easily cross the BBB, serving as an alternative pathway for SARS‐CoV‐2 neurotropism.

## 5 Mechanisms of EVs‐Related Neuropathology Following COVID‐19

It is widely acknowledged among scholars that neuroinflammation, ischemia, and hypoxic lesions caused by infarction or thrombosis of large and small cerebral vessels are the principal processes underlying CNS dysfunction associated with COVID‐19.^[^ ^132^ ^]^ These pathogenic changes in the brain may result from direct invasion of the neurological system by SARS‐CoV‐2. Additionally, SARS‐CoV‐2 infection leads to a systemic hypercoagulable state and cytokine storm, which further contribute to the activation of neuroinflammation and cerebral vascular thrombosis.^[^ ^15^, ^125^, ^133^ ^]^

### 5.1 EVs and Neuroinflammation

**Figure 4:**
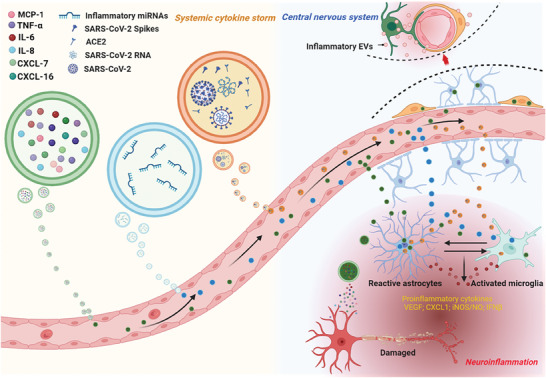
Modes and processes of EVs involvement in neuroinflammation. EVs contribute to the development of neuroinflammation, especially when a systemic cytokine storm breaks out after infection with SARS‐CoV‐2. First, with the assistance of extracellular vesicles, SARS‐CoV‐2 invades the CNS, infects nerve cells, and triggers neuroinflammation. Second, EVs deliver pro‐inflammatory mediators (microRNAs) to the CNS, which help transform nerve cells from resting to inflammatory states. In addition, following a systemic cytokine burst, EVs carry large amounts of peripherally produced inflammatory cytokines (MCP‐1, TNF‐α, IL‐6, IL‐8, CXCL‐7, CXCL‐16, VEGF) toward the CNS. On the one hand, blood‐brain barrier cells uptake the pro‐inflammatory cytokine‐delivering EVs and upregulate their inflammatory genes, further releasing inflammatory EVs to promote neuroinflammation. On the other hand, EVs carrying cytokines strut into the CNS triggering the activation of microglia and astrocytes. The activated microglia and astrocytes then release large amounts of pro‐inflammatory factors (such as IL‐1β, IL‐2, IL‐6, IL‐8, IL‐13, IL‐15, TNF‐α, CXCL‐1; VEGF, iNOS/NO), thereby promoting the progression of neuroinflammation. MCP‐1, monocyte chemotactic protein 1; TNF‐α, Tumor necrosis factor‐alpha; IL‐6, Interleukin‐8; IL‐8, CXCL‐1, chemokine (C‐X‐C motif) ligand 1; CXCL‐7, chemokine (C‐X‐C motif) ligand 7; CXCL‐16, chemokine (C‐X‐C motif) ligand 16; VEGF, Vascular endothelial growth factor; iNOS/NO, inducible nitric oxide synthase/Nitric oxide.

**Figure 5:**
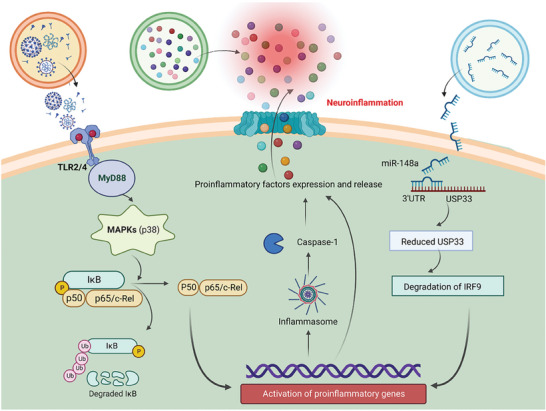
Potential mechanisms for the involvement of EVs in neuroinflammation. When EVs carrying SARS‐CoV‐2 and its components arrive in the CNS, SARS‐CoV‐2 and its components activate MYD88 via Toll‐like receptor 2/4 (TLR2/4), which then signals through NF‐κB to promote the expression and release of inflammatory cytokines. In addition, pro‐inflammatory miR‐148a transported by EVs to the CNS inhibits ubiquitin‐specific peptidase 33 (USP33) and downstream interferon regulatory factor 9 (IRF9), which in turn increases pro‐inflammatory gene expression. The pro‐inflammatory mediators produced by these processes, together with cytokines carried by EVs from the periphery into the CNS, lead to a burst of neuroinflammation.

Inevitably, infectious diseases provoke inflammation, and patients with COVID‐19 also exhibit signs of cytokine storm, which is a significant contributor to disease progression and mortality.^[^ ^134^ ^]^ Neurological manifestations observed in COVID‐19 patients are linked to systemic cytokine storm and neuroinflammation.^[^ ^135^ ^]^ The emergence of self‐reported neurological symptoms approximately four months after SARS‐CoV‐2 infection is associated with earlier instances of inflammation.^[^ ^136^ ^]^ Notably, individuals with SARS‐CoV‐2‐associated encephalitis have shown elevated levels of IL‐8, IL‐6, and TNF‐α in the CSF, suggesting that cytokine release syndrome is the primary inflammatory cause.^[^ ^137^ ^]^ It is important to recognize that SARS‐CoV‐2‐induced attacks on the nervous system can manifest as neuroinflammation, and it is necessary to comprehend how peripheral and systemic inflammation caused by COVID‐19 affects the nervous system (**Figure** **4**). Moreover, the potential neuroinflammation mechanisms of EVs involvement in the neuropathology of COVID‐19 have been preliminarily revealed (**Figure** **5**).

#### 5.1.1 EVs and Neuroinflammation Caused by SARS‐CoV‐2 Infection

Crucial contributors to neuroinflammation include various immune cells (lymphocytes and monocytes), resident cells (glial cells and glial cells), pro‐inflammatory cytokines, and inflammasomes.^[^ ^138^ ^]^ Studies in small samples indicate no evidence of immune cell infiltration or immunological manifestations in SARS‐CoV‐2‐containing brain regions or SARS‐CoV‐2‐positive CSF. This suggests that SARS‐CoV‐2 may not trigger the typical features of CNS viral infection.^[^ ^106^, ^139^ ^]^ However, this does not discount the possibility that the direct invasion of the nervous system by SARS‐CoV‐2 causes neuroinflammation. There are intriguing cues that support this speculation. First, in an autopsy series of 43 patients, brainstem inflammation was more severe in four cases (partially characterized by CD8+ T cell infiltration), and three of these four cases showed detectable SARS‐CoV‐2 protein or RNA in the brain.^[^ ^140^ ^]^ Schwabenland et al. demonstrated that viral antigens correlated with patterns of perivascular immune activation involving CD8^+^ and CD4^+^ T cells and microglial subsets.^[^ ^141^ ^]^ Second, astrocytes and microglia have been recognized as critical contributors to detrimental host responses in CNS disease.^[^ ^142^ ^]^ Reviewing the neuropathology of SARS‐CoV, Middle East Respiratory Syndrome Coronavirus (MERS‐CoV), and SARS‐CoV‐2, it becomes evident that these viruses not only infect astrocytes and microglia but also induce a pro‐inflammatory transformation, promoting neuroinflammation during viral infection.^[^ ^143^ ^]^ An in vitro study revealed that introducing the SARS‐CoV‐2 S protein into HEK‐293T cells produced EVs that were abundant in miR‐148a and miR‐590. These miRNAs, when taken up by human microglia cells, disrupted the expression of two important proteins: ubiquitin specific peptidase 33 (USP33), which plays a role in stabilizing its target protein, and interferon regulatory factor 9 (IRF9). Previous research has shown that IRF9 is crucial for maintaining CNS homeostasis, and its absence can lead to severe neurological damage in glial cultured cells due to overexpression of IFN‐α.^[^ ^144^ ^]^ Decreased levels of USP33 and IRF9 in microglia cells resulted in the activation of inflammatory pathways, such as TNF‐β, NF‐κB, and IFN‐β, leading to a cascade of neuroinflammation.^[^ ^145^ ^]^ Third, after intracranial inoculation of SARS‐CoV‐2 in monkeys, elevated levels of granulocyte colony‐stimulating factor (G‐CSF), interleukin (IL)‐2, IL‐8, IL‐13, IL‐15, and vascular endothelial growth factor (VEGF) were observed in the brain.^[^ ^107^ ^]^ Furthermore, SARS‐CoV‐2 nucleocapsides have been detected in brain samples along with the NOD‐like receptor pyrin domain‐related protein 3 (NLRP3) inflammasome, suggesting that the inflammasome may trigger an inflammatory form of programmed cell death (pyroptosis) in CNS during SARS‐CoV‐2 infection.^[^ ^146^ ^]^ While much of the in vivo evidence remains inferential, a wealth of in vitro evidence helps test this theory. Organoid studies have shown increased immune responses (e.g., innate immune responses) and inflammatory gene expression (e.g., TNF, INFγ) following SARS‐CoV‐2 infection.^[^ ^147^ ^]^ Similarly, SARS‐CoV‐2 can directly infect human microglia clone 3 (HMC3), causing it to exhibit the M1 phenotype, produce pro‐inflammatory cytokines such as IL‐6, IL‐1β, and TNF‐α, and inhibit the production of the anti‐inflammatory cytokine IL‐10.^[^ ^148^ ^]^ Exposure of microglia to SARS‐CoV‐2 S1 also activates microglia and promotes the release of inflammatory mediators, including TNF‐α, IL‐6, IL‐1β, chemokine (C‐X‐C motif) ligand 1 (CXCL1), NLRP3, and inducible nitric oxide synthase (iNOS)/nitric oxide (NO) pathway.^[^ ^149^ ^]^ Additionally, induction of neuroinflammation by SARS‐CoV‐2 S1 in microglia may be mediated by the TLR4/TLR2‐phosphorylation of p38 MAP Kinase (p38MAPK)/nuclear factor kappa B (NF‐κB) signaling pathway.^[^ ^149^ ^]^ Notably, EVs from patients with mild severity of COVID‐19 displayed higher levels of MHC class II‐antigen‐presenting proteins, which could interact with CD4+ T‐cells, stimulating their proliferation and activation.^[^ ^81b^ ^]^ Overexpression of MHC II, along with CD4 activation and invasion of the CNS, can trigger phagocytic conversion of brain myeloid cells mediated by IFN.^[^ ^150^ ^]^ This subsequent neuroinflammation and loss of dopaminergic neurons in the subthalamic nucleus pars compacta (SNpc) could potentially contribute to the development of neurodegenerative diseases, such as Parkinson's disease (PD).^[^ ^151^ ^]^

Given that direct invasion by SARS‐CoV‐2 can result in neuroinflammation, it is essential to understand the relationship between EVs and SARS‐CoV‐2‐induced neuroinflammation. EVs can act as a means of viral transmission between nerve cells,^[^ ^152^ ^]^ thereby facilitating the spread and impact of the inflammatory effects caused by SARS‐CoV‐2 infection. Additionally, virus‐infected neural cells and activated immune cells continually release EVs containing pro‐inflammatory mediators, which contribute to persistent neuroinflammation.^[^ ^152^, ^153^ ^]^ Nonetheless, limited in vivo evidence conclusively demonstrates neuroinflammation induced by EV released from individuals infected with SARS‐CoV‐2. At the cellular level, however, findings suggest that cells transfected with SARS‐CoV‐2 S1 can release miRNA‐enriched EVs. When internalized by human microglia, these EVs effectively activate three control inflammatory pathways, namely NF‐κB, TNF‐α, and interferon‐beta (IFN‐β).^[^ ^150^ ^]^ To shed light on the role of EVs in neuroinflammation driven directly by SARS‐CoV‐2, It is crucial to focus on EVs (donor EVs) released from various isolated nerve cells with SARS‐CoV‐2 infection and observe EVs (recipient EVs) released from uninfected nerve cells treated with donor EVs. An omics study or analysis of the inflammatory pathway involved in this process would provide valuable insights.

#### 5.1.2 EVs and Neuroinflammation Resulted from Systemic Inflammation

Elevated levels of pro‐inflammatory cytokines orchestrate a storm, which is frequently observed in patients with COVID‐19 pneumonia.^[^ ^154^ ^]^ Following this storm, significant and persistent neuroinflammation is observed even in the absence of detectable SARS‐CoV‐2 in the brains of COVID‐19 patients, suggesting that factors other than the direct effects of the virus on the nervous system contribute to brain inflammation.^[^ ^117a^ ^]^ Exosomes containing SARS‐CoV‐2 components, RNA, and neuroregulatory molecules induced by the virus have the ability to reach neuroanatomical regions that are typically difficult to access. These regions include the olfactory bulb,^[^ ^126b^ ^]^ hypothalamus,^[^ ^155^ ^]^ DMV, and brainstem.^[^ ^156^ ^]^ COVID‐19 patients with encephalitis or encephalopathy exhibited high levels of IL‐6 and IL‐8 in CSF without any present of SARS‐CoV‐2.^[^ ^157^ ^]^ Indeed, SARS‐CoV‐2 infection prompts the activation of numerous immune cells in the human body, initiating a cascade of reactions that lead to a fatal systemic hyperinflammatory response, also known as a cytokine storm. This cytokine storm involves the release and spread of various proinflammatory cytokines and chemokines throughout the body, including IL‐1α, IL‐1β, IL‐2, IL‐6, IL‐7/8/9/10, C‐C motif chemokine ligand 2/monocyte chemotactic protein 1 (CCL2/MCP‐1), CXCL‐10, granulocyte‐macrophage colony‐stimulating factor (GM‐CSF), VEGF, macrophage inflammatory protein 1‐alpha/‐beta (MIP‐1‐α/β), and TNF.^[^ ^134^, ^158^ ^]^ Unsurprisingly, this systemic cytokine storm wreaks havoc on the nervous system. COVID‐19 patients with neurological involvement exhibit significantly higher markers of systemic inflammation compared to those without neurological injury.^[^ ^159^ ^]^ Moreover, research indicates that elevated levels of systemic cytokines such as IL‐6, IL‐8, and IL‐1 are associated with subsequent neuro‐axonal impairment in COVID‐19 individuals.^[^ ^160^ ^]^ Therefore, it can be inferred that such a potent peripheral or systemic cytokine storm inevitably leads to adverse effects on the neurological system, resulting in neuroinflammation.

To the best of our knowledge, peripheral cytokines may contribute to neuroinflammation through two mechanisms. First, pro‐inflammatory mediators can cross the BBB and induce neuropathy in COVID‐19 patients.^[^ ^132^, ^161^ ^]^ It is worth noting that EVs possess the inherent ability to traverse the BBB, and EVs produced under inflammatory conditions can also trigger neuroinflammation in the brain.^[^ ^162^ ^]^ Cytokines and chemokines can be packaged within EVs, released from cells, and then transported to specified locations, including the brain, to carry out vital biological functions.^[^ ^163^ ^]^ In essence, EVs serve as a mode of remote delivery for cytokines and chemokines, playing a crucial role in the amplification of local inflammation and the propagation of systemic inflammation during disease.^[^ ^164^ ^]^ A proteomic analysis of circulating EVs from COVID‐19 patients unveiled elevated levels of numerous inflammatory, immune response, and coagulation proteins, such as CXCL‐7 and IL‐6.^[^ ^82^, ^165^ ^]^ Plasma‐derived EVs from COVID‐19 patients were found to carry various cytokines (TNF‐α, IL‐6, IL‐8) and chemokines (MCP‐1, CXCL16).^[^ ^166^ ^]^ From a neurological perspective, when these inflammatory mediators additional cytokines are produced, and the serum cytokines enter the CNS, additional cytokines are produced, and a combination of serum cytokines and newly generated cytokines contribute to neuroinflammation.^[^ ^167^ ^]^ Notably, the cytokine content within plasma‐derived EVs may contribute to the persistence of post‐stroke neuroinflammation and correlate with a poor prognosis.^[^ ^168^ ^]^ EV from COVID‐19 patients can stimulate distant organ cells to produce inflammatory signals like TNF‐, IL‐6, and NLRP3 inflammatory bodies.^[^ ^169^ ^]^ Therefore, it is tempting to speculate that EVs carry cytokines or chemokines to invade the CNS, leading to neuroinflammation and neuropathic damage in COVID‐19. Furthermore, due to the SARS‐CoV‐2‐induced inflammatory storm, a substantial amount of inflammatory miRNAs is produced.^[^ ^170^ ^]^

As the main transport mechanism for free miRNAs, EVs can transmit a large number of inflammatory signals by carrying miRNAs to regulate gene expression and cellular function at a distance.^[^ ^171^ ^]^ This suggests that the circulating inflammatory miRNAs detected thus far might be transported into the brain via EVs and subsequently impact COVID‐19‐related CNS damage. However, current evidence is lacking in this regard. Alternatively, systemic inflammatory mediators in the peripheral circulation may not directly enter the CNS but instead act on the BBB. BBB cells utilize EVs as relay mechanisms to transmit inflammatory signals, overwhelming the CNS. Increasing evidence suggests that peripheral inflammation leads to increased EVs released by choroid plexus epithelial cells into the CSF, which is subsequently taken up by brain cells, further inducing CNS inflammation.^[^ ^172^ ^]^ Similarly, in response to peripheral inflammation, inflammatory genes in barrier cells of the choroid plexus are broadly upregulated in COVID‐19 cases, transmitting inflammation from the periphery to the brain.^[^ ^117c^ ^]^ Astrocytes, a type of BBB cells, are highly sensitive to pro‐inflammatory cytokines, and IL‐1‐activated astrocytes released numerous EVs containing specific miRNAs that can serve as indicators of neuroinflammation.^[^ ^173^ ^]^ Additionally, it is widely acknowledged that the integrity and tight junctions of the BBB are severely compromised in the presence of COVID‐19 ^[^ ^174^ ^]^, and BBB damage is closely associated with neuroinflammation. However, the exact mechanisms by which these EVs carrying inflammatory mediators disrupt the BBB remain unknown, and further evidence is necessary to confirm this hypothesis. To conclude, EVs play an important role in the interaction between systemic inflammation on neuroinflammation, but additional evidence is required to fully understand this mechanism.

### 5.2 EVs and Cerebral Thrombosis

**Figure 6:**
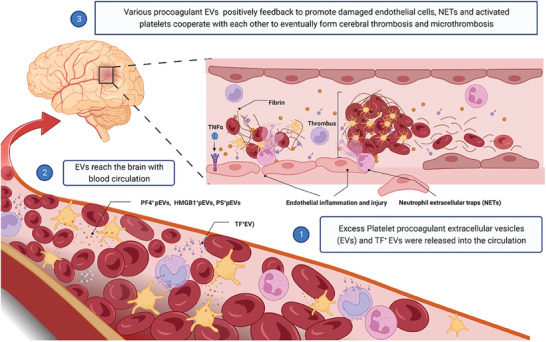
EVs and cerebral thrombosis. Due to direct infection with SARS‐CoV2 or a hyperinflammatory state caused by SARS‐CoV2 infection, activated platelets release large amounts of procoagulant platelet‐derived EVs (pEVs) such as high mobility group frame 1 protein (HMGB1)^+^pEVs, platelet factor 4 (PF4)^+^pEVs, and phosphatidylserine (PS)^+^pEVs while inducing the release of tissue factor (TF)^+^EVs from monocytes. These procoagulant EVs reach the brain through blood circulation, where they work together with damaged endothelial cells and neutrophil extracellular traps (NETs) to eventually lead to the formation of immune thrombi in cerebral circulation or microcirculation. HMGB1, high mobility group frame 1 protein; NETs, neutrophil extracellular traps; PF4, platelet factor 4; PS, phosphatidylserine; pEVs, platelet‐derived extracellular vesicles; TF, tissue factor.

A systematic review indicated that the incidence of acute ischemic stroke (AIS), cerebral hemorrhage (CH), subarachnoid hemorrhage (SAH), and cerebral venous thrombosis (CVT) in COVID‐19 patients with cerebrovascular complications was 71.2%, 19.8%, 1.8%, and 7.2% respectively. This implies that nearly 80% of cerebrovascular events were attributed to ischemic cerebrovascular events.^[^ ^175^ ^]^ The high mortality rate associated with COVID‐19‐related cerebrovascular events underscores the importance of understanding the underlying mechanisms. Although the mechanisms responsible for cerebral ischemia in COVID‐19 patients are still unknown, growing evidence suggests that vascular endothelial injury, persistence hypercoagulability, and hyperinflammation make COVID‐19 patients more susceptible to thrombotic events at both the microvascular and macrovascular levels.^[^ ^176^ ^]^ Some scholars have amassed substantial evidence suggesting that circulating EVs act as risk factors by targeting the already fragile microcirculation of the COVID‐19 brain, thereby becoming a new harmful pathological mechanism mediating COVID‐19‐related CSVD.^[^ ^177^ ^]^ Therefore, we aimed to discuss the role of EVs in cerebral thrombosis and micro‐thrombosis based on the three factors mentioned above. Initially, endothelial cells (ECs) are one of the target cell types for SARS‐CoV‐2 infection. SARS‐CoV‐2 promotes endothelial damage in various organs by inducing autophagy and apoptosis and activating an inflammatory phenotype in ECs.^[^ ^178^ ^]^ Although there is no definitive proof, it has been hypothesized that EVs may be involved in this endothelial damage due to their ability to carry viruses or viral components. Plasma EVs from COVID‐19 patients trigger the NLRP3 inflammasome in ECs and cause EC death through a phosphatidylserine (PS)‐dependent mechanism.^[^ ^169^, ^179^ ^]^ In addition, non‐viral components carried by EVs may also play a role. Two specific microRNAs (miR‐145 and miR‐885) contained in circulating EVs are functionally involved in thromboembolic events in COVID‐19 by interfering with ECs and COVID‐19.^[^ ^180^ ^]^ Notably, miR‐24, which is expressed in human brain ECs, is associated with cerebrovascular (CBV) manifestations of COVID‐19 through ECs‐derived EV miR‐24 in plasma.^[^ ^99^ ^]^ Second, in terms of the hypercoagulable state, abnormal coagulation system resulting from EVs in viral infections such as hantavirus, HIV, and influenza A (H1N1) virus promote thrombosis.^[^ ^181^ ^]^ The well‐documented role of EVs in coagulation abnormalities and thrombotic events also applies to COVID‐19.^[^ ^182^ ^]^ Plasma EVs from SARS‐CoV‐2 infected patients often exhibit procoagulant activity, thereby initiating the systemic coagulation cascade.^[^ ^183^ ^]^ These EVs have been recognized as novel circulating biomarkers in assessing the hypercoagulable state and thrombosis in COVID‐19.^[^ ^184^ ^]^ The components of thrombosis involving coagulation factors are divided into circulating components (platelets and their activating factors and some coagulation factors) and vessel wall components consisting of tissue factor (TF), damaged endothelium, and exposed collagen. These two components are closely associated with EVs in COVID‐19. Platelets have a central role in the thrombotic inflammatory response, and platelet‐derived EVs (pEVs) have procoagulant activity on their surface ≈50 to 100 times higher than activated platelets.^[^ ^185^ ^]^ Some academics have observed that activated platelets release pEVs with procoagulant potential, which can excessively activate blood coagulation and lead to thrombosis of COVID‐19.^[^ ^186^ ^]^ Importantly, pEVs continue to increase 30 days after patients are discharged, indicating that platelet activation with EVs persists well beyond the acute phase of SARS‐CoV‐2 infection.^[^ ^187^ ^]^ This raises the possibility that pEVs may be responsible for long‐term complications (e.g. long COVID syndrome) in COVID‐19 patients. A procoagulant protein called TF is present on the surface of EVs released by activated host cells like monocytes. Similarly, elevated levels of EVTF activity and an increase in the number of TF^+^ EVs were observed in the circulation of COVID‐19 patients, which also contributes to thrombosis in these patients.^[^ ^181^, ^188^ ^]^ Furthermore, EVTF activity was significantly increased in critically ill patients (median, 231 [25th to 75th percentiles, 39‐761]) fM, and its activity ≥0.565 pg mL^−1^ was associated with higher mortality in moderate patients (median, 25 [25th to 75th percentiles, 12‐59]) fM.^[^ ^188^, ^189^ ^]^ Lastly, COVID‐19‐associated thrombosis is essentially immunothrombotic, meaning that hyperinflammation is also involved in the development of COVID‐19‐related immunothrombosis by interacting with the hypercoagulable state and EC dysfunction. In the inflammatory environment caused by SARS‐CoV‐2 infection, high concentrations of inflammatory mediators lead to neutrophil activation and the release of neutrophil extracellular traps (NETs).^[^ ^190^ ^]^ Not only can NETs, in turn, act as pro‐inflammatory inducers,^[^ ^191^ ^]^ but they are also considered important promoters of immune‐mediated thrombosis in COVID‐19.^[^ ^192^ ^]^ Reports indicate that neutrophils dominate the immunological profile of cerebral thrombi in COVID‐19 stroke patients,^[^ ^193^ ^]^ and NETs are prevalent in cerebral thrombi with a cardioembolic origin.^[^ ^194^ ^]^ ICAM‐1 facilitates the attachment of vascular ECs and leukocytes, thereby promoting wall thrombi development. Thus, the high levels of ICAM‐1^+^EV observed in the blood of COVID‐19 patients may directly contribute to immune thrombosis.^[^ ^195^ ^]^ PS is often linked to thrombo‐inflammation in COVID‐19, and circulating EVs in COVID‐19 patients induce an increase in PS‐dependent neutrophil adhesion.^[^ ^179^ ^]^ Peripheral blood mononuclear cells (PBMC) from COVID‐19 patients exhibited high surface exposure to PS, which correlates with the presence of PS^+^EVs and the severity of the disease. Notably, PS^+^EVs in PBMC are PS^+^CD4^1+^pEVs.^[^ ^196^ ^]^ Additionally, platelets and neutrophils frequently interact in the formation of immunothrombosis. High mobility group frame 1 protein (HMGB1)^+^pEVs and platelet factor 4 (PF4)^+^pEVs may promote neutrophil activation and the formation of NETs in COVID‐19.^[^ ^186^, ^197^ ^]^ Furthermore, pEVs have been shown to promote NETs generation during SARS‐CoV‐2 infection through TLR2 and CLEC5A‐dependent mechanisms.^[^ ^198^ ^]^ In conclusion, SARS‐CoV2 infection or pro‐inflammatory mediators activated platelets, damaged endothelial cells, and immune cells, resulting in the release of large volumes of pro‐coagulant EVs. This surge of EVs contributes to the collaboration of thrombogenic factors, leading to the development of a microvascular coagulation storm that ultimately results in cerebral thrombosis and microthrombosis in COVID‐19 (**Figure** **6**).

## 6 The Promising Applications for EVs in COVID‐19‐Related Neurological Complications

**Figure 7:**
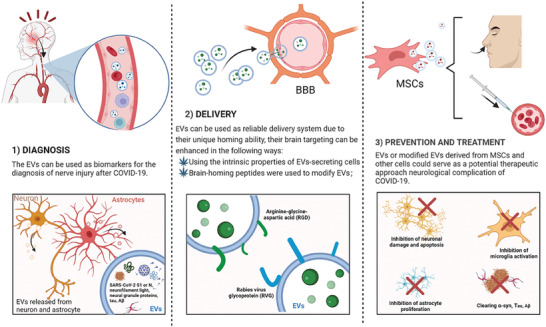
The promising application for EVs in COVID‐19‐related neurological complications. The NDEV containing N protein of SARS‐CoV‐2, Aβ, neurofilament light, neural granule proteins, tau, and ADEV exosomes are promising targets for COVID‐19 diagnostic tools. Due to their inherent homing properties, EVs from MSCs and other cells are expected to enter the BBB for therapeutic purposes in the CNS. The modification of EVs with a series of brain‐homing peptides such as c(RGDyK), RVG, etc., has made EVs even more reliable brain‐targeted transporter systems. Finally, EVs have been shown to serve as effective vaccine delivery platforms, particularly those of MSC origin. They may exert central protective effects by removing degenerative proteins, inhibiting microglial activation, suppressing astroglial proliferation, and rescuing neuronal injury and death. NDEV: neuron‐derived EVs; ADEV: astrocyte‐derived EVs; MSCs: Mesenchymal stem cells; c(RGDyK): arginine‐glycine‐aspartic acid (RGD)‐4C peptide and cyclic arginine‐glycine‐aspartic acid‐tyrosine‐lysine; RVG: rabies virus glycoprotein.

Extensive research during the COVID‐19 pandemic has primarily focused on the potential of EVs in diagnosis, prognosis, and therapy. With their ample cargo and abundant presence in body fluids, particularly the blood, EVs offer reliable and convenient diagnostic biomarkers. Furthermore, SARS‐CoV‐2 infection triggers the release of EVs from different sources, including platelets. Identifying specific markers in these EVs within the bloodstream holds promise as prognostic indicators of disease severity. EVs possess biocompatibility, inherent material transport properties, editable targeting, long‐term recycling ability, and inheritance of parental cell properties, positioning them as highly promising nanocarriers for drug delivery and active biologics. Notably, clinical trials have highlighted the therapeutic potential of EVs derived from mesenchymal stem cells for severely affected patients, while EVs sourced from convalescent plasma showcase multiple efficacies. Additionally, EVs loaded with messenger RNA (mRNA) have demonstrated their superiority to lipid nanoparticles in enabling safe delivery and induction of SARS‐CoV‐2 immunity, encoding immunogenic viral proteins. Moreover, EVs exhibit potential as nano‐delivery systems for microRNA, effectively attenuating cytokine storms and preventing organ failure progression in COVID‐19 patients. Lastly, the utilization of EVs derived from engineered pathogens offers a practical and straightforward vaccination strategy against COVID‐19(**Figure** **7**).

### 6.1 EVs as Biomarkers for the Diagnosis of Neurological Injury Following COVID‐19

In a significant contribution, Elletra et al. employed proteomics to confirm the presence of COVID‐19 in the exosomes of patients, demonstrating their role in facilitating infection spread across various cells in the body.^[^ ^82^ ^]^ From another perspective, these harmful EVs could be effectively exploited as predictors of COVID‐19 brain damage with appropriate detection.^[^ ^199^ ^]^ Utilizing exosomal detection of the virus for COVID‐19 diagnosis can provide insights into disease severity and accurate detection in symptomatic patients. For instance, mild COVID‐19 patients have more SARS‐CoV‐2‐S‐derived fragments in exosomes than severe patients and can better activate immune responses.^[^ ^81b^ ^]^ Recent studies have shown the presence of SARS‐CoV‐2 viral proteins in brain‐derived exosomes, which could contribute to the spread and severity of SARS‐CoV‐2. Analysis of neuron‐derived EVs (NDEV) and astrocyte‐derived EVs (ADEV) in the blood plasma of COVID‐19 patients revealed significantly higher levels of critical SARS‐CoV‐2 structural proteins (S1 and N) compared to healthy individuals. Notably, the levels of N protein in ADEV and NDEV could differentiate patients with long COVID‐19 and neuropsychiatric symptoms from those with long COVID‐19 but no neuropsychiatric complications, as well as from those who had recovered from COVID‐19 without experiencing long‐term symptoms.^[^ ^98^ ^]^ Furthermore, Sun et al. found that neurodegenerative proteins such as, for example, Aβ, neurofilament light, neural granule proteins, total tau, and p‐T181‐tau were significantly increased in neuronal EV in almost all participants recovering from COVID‐19 for one month to three months.^[^ ^97^ ^]^ Although it is unknown whether such protein markers of neuronal dysfunction in EV are long‐lasting, these could all be potential plasma biomarkers of neuroinflammation or neuronal damage following COVID‐19. It is plausible to hypothesize that the exosomal cargo related to COVID‐19, which contains persistent SARS‐CoV‐2 viral components or neurodegeneration‐related proteins, could potentially contribute to the neurodegenerative development and manifestation, both during the acute phase and possibly even after COVID‐19. Exosomes carrying components of SARS‐CoV‐2 can be diagnosed using fluorescent or bioluminescent dyes to label the protein of interest. While exosomes present a promising target for COVID‐19 diagnostic tools, it is crucial to identify standard biomarkers for their detection and develop strategies based on EVs for diagnostic tool development.^[^ ^200^ ^]^ Notably, convalescent plasma‐derived exosomes hold potential in both diagnosis and therapeutics for COVID‐19.

### 6.2 EVs, the Reliable Delivery System for COVID‐19 Central Neuropathology

Due to their origin and lipophilic properties, EVs possess a unique advantage in crossing the BBB, making them excellent candidates for delivering therapeutic compounds to brain tissue. Besides, EVs have a unique homing ability. Studies in various models of neurodegenerative and neurodevelopmental disorders have shown that therapeutic EVs can reach diseased brain regions and be taken up by neurons through their neuroinflammation‐driven homing mechanism.^[^ ^201^ ^]^ Based on this premise, it is hypothesized that natural EVs, especially MSC‐EVs, hold promise for treating neuropathy in COVID‐19 due to their inherent homing property. In addition, EVs serve as highly efficient carriers for delivering biologically active proteins or drugs, making them an attractive approach for treating central disorders, including COVID‐19‐related neurological complications.^[^ ^202^ ^]^ Hence, it is necessary to discuss methods for enhancing the brain‐targeting capability of EVs. The intrinsic properties of EVs‐secreting cells can be leveraged to enhance EVs targeting. For instance, macrophage exosomes use integrins such as lymphocyte function‐associated antigen 1 (LFA‐1) and ICAM‐1, as well as carbohydrate‐binding C‐type lectin receptors, to facilitate interaction with the BBB, leading to increased brain accumulation during neuroinflammation.^[^ ^124^ ^]^ However, this natural approach is not specific, and EVs may accumulate in organs other than the nervous system. Surface engineering of EVs through genetic modification of EV‐secreting cells or direct modification of EV surface components or contents represents an effective approach to achieving and enhancing EV brain‐targeting capabilities.^[^ ^203^ ^]^ A series of brain‐homing peptides that target specific regions in the ischemic brain have been developed based on EV surface modifications, such as the arginine‐glycine‐aspartic acid (RGD)‐4C peptide and cyclic arginine‐glycine‐aspartic acid‐tyrosine‐lysine (c(RGDyK) peptide.^[^ ^204^ ^]^ Additionally, there is a modification using the rabies virus glycoprotein (RVG) peptide, which targets neural cells through genetic modification of EV‐secreting cells.^[^ ^205^ ^]^ The development of these brain‐homing peptides will significantly facilitate the application of EVs in the future treatment of COVID‐19‐related neurological complications.

The mode of administration for EVs may also influence their effects. A study by Zhao demonstrated that inhalation, rather than intravenous injection, is the optimal administration route for MSC‐EVs in treating acute lung injury.^[^ ^206^ ^]^ Interestingly, intranasal administration of therapeutic MSC‐EVs prevents their entry into the systemic circulation, as they are cleared, primarily by the liver and spleen, when administered through other systemic routes.^[^ ^207^ ^]^ Instead, this intranasal route enables EVs to accumulate better in the brain and exert neuroprotective effects through a special nasal‐brain connection.^[^ ^207^, ^208^ ^]^ Thus. EVs have shown significant therapeutic potential in COVID‐19‐related neuropathy, and both the International Society for Cell and Gene Therapy (ISCT) and the International Society for Extracellular Vesicles (ISEV) have encouraged their development under strict regulation for the treatment of COVID‐19.^[^ ^18a^ ^]^ However, no biological tool is perfect, and these pieces of evidence and directions provide hope for realizing the therapeutic role of EVs in the future. It is worth believing that with the continuous clarification of the pathogenesis of COVID‐19, the development of effective drugs for COVID‐19 treatment, and the ongoing research on EVs, these vesicles are expected to serve as a favorable biological tool to fulfill their therapeutic potential in COVID‐19‐related neuropathy in the future.

### 6.3 EVs, the Promising Prevention and Treatment Mediator for COVID‐19 Central Neuropathology

A pandemic of any infectious disease inspires a desire that leads to a vaccine. Fundamental to the prevention of COVID‐19 neurological complications is the prevention of SARS‐CoV‐2 infection and invasion. EVs are considered a promising viral vaccine carrier. Recent studies have highlighted EVs‐based vaccines' efficacy, biocompatibility, and safety against COVID‐19.^[^ ^209^ ^]^ The study proved the effectiveness of exosomes for inhalation and showed superior immunological advantages, allowing for faster clearance of the virus.^[^ ^210^ ^]^ In fact, EV‐ACE2 was 60‐ to 80‐fold more effective in preventing pseudotyped and true SARS‐CoV‐2 infections compared to vesicle‐free recombinant human ACE2 (rhACE2).^[^ ^211^ ^]^ With the passing of the COVID‐19 pandemic, This EVs‐based viral vaccine is critical in the preventive role of other viruses. Knowledge of EV‐based vaccine development against different viruses has been summarized, showing that immunization with EV can activate the immune response to a wide range of viral infections, such as porcine reproductive and respiratory syndrome virus(PRRSV), lymphocytic choroid plexus meningitis virus (LCMV)and Marek's disease virus (MDV).^[^ ^212^ ^]^ In conclusion, this evidence encourages us to explore in depth EVs‐based vaccines. We believe that this efficient vaccine strategy will show great potential for preventing viral infections in the face of new viral infection crises in the future.

Some scholars have proposed using EV as a drug carrier or biological mediator for treating neurological complications of COVID‐19 and other viruses.^[^ ^213^ ^]^ MSCs, known for their multipotency, play a crucial role in regenerative medicine by differentiating into various mesenchymal tissues like bone, tendon, adipose tissue, marrow stroma, and muscle. EVs derived from MSCs have emerged as a promising approach for protecting, repairing, and regenerating organs in SARS‐CoV‐2 patients.^[^ ^214^ ^]^ Given the common and devastating neurological complication during SARS‐CoV‐2 infection, the use of MSCs‐EVs has shown promising outcomes in early studies. Additionally, the positive effects of MSC‐EVs to reduce neuroinflammation in animal stroke models have been established. Therefore, MSC‐EVs can be considered a supportive treatment in COVID‐19 patients, with the aim of mitigating both primary neuroinflammation caused by direct CNS infection and secondary neuroinflammation induced by cytokine storm. Additionally, human umbilical cord origin MSCs‐derived‐EVs possess anti‐inflammatory properties, inhibiting the activation of A1 astrocytes by regulating Nrf2/NF‐κB signaling.^[^ ^215^ ^]^ Similarly, MSC‐EVs have demonstrated the ability to suppress neuronal apoptosis and facilitate the recovery of spinal cord function following CNS injury through the activation of Wnt/β‐catenin signaling.^[^ ^216^ ^]^ These findings suggest that these EVs could serve as a potential therapeutic approach for inflammation‐related neurological disorders. Furthermore, MSC‐derived EVs have been shown to support functional restoration and the remodeling of neurovascular abnormalities in a preclinical model of cerebral hemorrhage stroke.^[^ ^217^ ^]^ On the other hand, the beneficial effects of MSC‐EVs are attributed to their ability to counteract the cytokine storm by inhibiting proinflammatory factors including IL‐1β, IL‐6, IL‐17, TNF, and IFN‐γ, while promoting the release of anti‐inflammatory factors such as IL‐4, IL‐10, and TGF‐β.^[^ ^218^ ^]^ Exosomal active mitochondria mediate the protective effects by enhancing oxidative phosphorylation, facilitating the transition of macrophage polarization from M1 to M2 state,^[^ ^219^ ^]^ and promoting a shift in the ratio of regulatory to effector T cells.^[^ ^220^ ^]^ Additionally, convalescent plasma is enriched with trillions of EVs that function as immunomodulators. These EVs contain protein material derived from SARS‐CoV‐2, which has the potential to enhance the immune response.^[^ ^221^ ^]^ To date, there have been three clinical studies focusing on MSCs or MSC‐EVs for central system aspects such as the treatment of fatigue (**Table** **2**). Unfortunately, however, no EV‐based clinical trial studies exist for other neurological manifestations in COVID‐19 patients. In conclusion, EVs have the potential to contribute to the recovery of neurological abnormalities through a variety of mechanisms, including facilitating neurogenesis, maintaining blood‐brain barrier (BBB) integrity, suppressing inflammation and apoptosis, and ultimately limiting disease progression.^[^ ^222^ ^]^ At the same time, the high incidence and long‐term effects of neurological manifestations urgently require the evaluation of the clinical efficacy of such cell‐free therapies, focusing on the prognosis of neurological deficits.

**Table 2:** Delivery and Therapeutic role of MSCs/EVs in COVID‐19‐related central neuropathology.

Diseases	EVs	Model	Injection type	Outcomes/Purpose	Ref/NCT ID
**COVID‐19**					
COVID‐19, postviral syndrome, dyspnea	EVs from Bone marrow mesenchymal stem cells (BM‐MSC‐EVs)	Human	Intravenous	Outcome: ‐ Status: Not yet recruiting	NCT05116761
COVID‐19	Cells from MSCs	Human	Intravenous	Patients’ symptoms of weakness alleviated 2–4 days after transplantation	[223]
COVID‐19	Cells from human umbilical cord‑derived (hucMSCs)	Human	Intravenous	Less fatigue and listlessness	[224]
**CNS inflammatory disorders**					
LPS‐induced brain inflammation	Exosomes from EL‐4 encapsulating curcumin (Exo‐cur)	Mice	Intranasal	The number of activated inflammatory microglia in the brain was significantly reduced; microglial apoptosis was induced	[127b]
Experimental autoimmune encephalomyelitis (EAE)	Exosomes from EL‐4 encapsulating curcumin (Exo‐cur)	Mice	Intranasal	EVs treatment resulted in a significant reduction in disease severity and a substantial reduction in activated microglia	[127b]
EAE	Exosomes from dendritic cells (DCs) secreting TGF‐β1 (sTGF‐β1‐EXOs)	Mice	Intravenous	mTGF‐β1‐EXOs possessed a potent immunoregulatory activity and were effective in inhibiting the progression of EAE	[225]
Japanese encephalitis virus (JEV)	BM‐MSC‐EVs	Mice	intraperitoneal	EVs attenuate viral replication, upregulate interferon‐stimulated genes, enhance immune response, delay symptoms and death in EAE mice, prolong survival, and accelerate neurogenesis in primary neuronal stem cells	[226]
**Cerebrovascular disease**					
Stroke	multipotent MSCs‐EVs	Human	Intravenous	Outcome: ‐ Status: Recruiting	NCT03384433
Stroke	EVs from adipose‐derived MSCs (ADMSCs)	Mice	Intravenous	The infarct size was reduced and increasing neurological recovery	[227]
Stroke	EVs from human placenta mesenchymal stem cells (hPMSC‐EVs)	Mice	Intravenous	Protecting the ipsilateral hemisphere from ischemic injury	[228]
Stroke	BM‐MSCs‐derived exosomes containing miR‐138‐5p	Mice	‐	Alleviating neuron injury in ischemic mice	[229]
Stroke	Exosomes from BM‐MSCs modified with rabies virus glycoprotein (RVG) Lamp2b, loaded with miR‐124 mimics	Mice	Intravenous	Promoting cortical neural progenitors to obtain neuronal identity, protecting against ischemic injury by robust cortical neurogenesis, and inhibiting astrocyte proliferation	[230]
Stroke	Exosomes from primary mouse neural stem cell (NSC)	Mice	Intravenous	Reducing infarct volumes	[231]
Stroke	M2 microglia‐derived exosomes		Intravenous	Attenuating ischemic brain injury and promoting neuronal survival via exosomal miR‐124 and USP14	[232]
Stroke	Cocultures of regular and oxygen‐glucose‐deprived MSC‐EVs	Rat	Intravenous	Reducing the infarct size and ipsilateral hemisphere swelling, preserving the neurological function, and facilitating the recovery of stroke‐induced rats	[233]
Stroke	Astrocyte‐derived exosomes (AS‐Exo)	Mice	Intravenous	Ameliorating neuronal damage through regulating autophagy	[234]
Stroke	miR‐30d‐5p‐overexpressing exosomes secreted from ADMSCs	Mice	Intravenous	Preventing cerebral injury by inhibiting autophagy‐mediated microglial polarization to M1	[235]
Stroke	human urine‐derived stem cells (USC‐Exos)	Rat	Intravenous	Enhancing neurogenesis and alleviated neurological deficits in post‐ischaemic stroke rats	[236]
Stroke	Macrophage‐derived exosomes containing Edaravone (Exo + Edv)	Rat	Intravenous	Reducing the death of neuronal cells and promoting the polarization of microglia from M1 to M2	[237]
Stroke	Baicalin‐loaded macrophage‐derived exosomes (Exo‐BA)	Rat	Intravenous	Exo‐BA displayed better brain targeting ability than free BA; Compared with free BA, Exo‐BA significantly reduced the generation of reactive oxygen species and activated the Nrf2/HO‐1 pathway in neurons	[238]
Stroke	Arginine‐glycine‐aspartic acid (RGD)‐C1C2‐ modified EVs (RGD‐EV) derived from neural progenitor cell	Mice	Intravenous	The RGD‐EV targeted the lesion region of the ischemic brain and resulted in a strong suppression of the inflammatory response	[204b]
Stroke	Engineered c(RGDyK)‐conjugated exosomes (cRGD‐Exo) loaded with curcumin from MSCs	Mice	Intravenous	cRGD‐Exo) targeted the lesion region of the ischemic brain, resulting in a strong suppression of the inflammatory response and cellular apoptosis in the lesion region	[204a]
Stroke	RGD‐Exo loaded with miR‐210 (RGD‐exo:miR‐210) from MSCs	Mice	Intravenous	The RGD‐exo:miR‐210 targets the lesion region of the ischemic brain, and upregulated expressions of integrin β3, vascular endothelial growth factor (VEGF), and CD34	[239]
Stroke	BM‐MSC‐EVs loaded with enkephalin(tar‐exo‐enkephalin)	Rats	Intravenous	tar‐exo‐enkephalin crossed the blood‐brain barrier (BBB) and decreased the levels of LDH, p53, caspase‐3, and NO.	[240]
Stroke	RVG‐exosomes from Human HEK‐293 cells loaded with nerve growth factor (NGF@Exo^RVG^)	Mice	Intravenous	Being highly stable for preservation and function efficiently for a long time in vivo, reducing inflammation, promoting cell survival, and increasing the population of doublecortin‐positive cells	[241]
**Neurodegeneration**					
AD	EVs from Allogenic ADMSCs	Human	intranasal	Status: Recruiting	NCT04388982
Dementias	Exosomes from healthy, full‐term Cesarean section amniotic fluid	Human	Intravenous	Outcome: ‐ Status: Suspended, pending COVID‐19 pandemic	NCT04202770
AD	BM‐MSC‐EVs	Mice	Intranasal	Improving cognition, reducing Aβ plaque	[242]
AD	hucMSCs‐EVs	Mice	Intravenous	Repairing cognitive dysfunctions, helping to clear Aβ deposition, and modulating the activation of microglia in the brains of the mice to alleviate neuroinflammation	[243]
AD	microglial exosomes loaded with miR‐124‐3p	Mice	Intravenous	Transferred into hippocampal neurons and alleviated neurodegeneration by targeting the Rela/ApoE signaling pathway	[244]
AD	RVG‐modified MSC‐EVs	Mice	Intracerebral	Improving cortical and hippocampal targeting and cognition, reducing plaque deposition, Aβ level, reduced astrocyte activation, and inflammation	[245]
AD	EVs loaded with miR‐29a/b from rat BM‐MSCs and HEK‐293T cells	Rat	Intracerebral	Improving cognition, and reducing the pathological effects of amyloid‐β (Aβ)	[246]
AD	MSC‐EVs	Mice	Intracerebroventricular	Stimulating neurogenesis in the subventricular zone and alleviated beta amyloid 1‐42‐induced cognitive impairment	[247]
AD	Mouse/Rat NSCs EVs	Mice	Intracerebroventricular	Abolishing Aβo‐induced suppression of LTP and subsequent memory deficits, enhancing mitochondrial function, SIRT1 activation, synaptic activity, decreased inflammatory response	[248]
AD	Exo‐cur from Mouse macrophage	Mice	Intraperitoneal	Exo‐cur enhanced cur accumulation in the hippocampus, improved cognition, and reduced neuronal damage	[249]
AD	RVG‐modified exosomes loaded with GAPDH siRNA from mouse DCs	Mice	Intravenous	Knockdown of BACE1 mRNA and protein, reducing Aβ protein and alleviating AD symptoms	[205]
AD	plasma exosomes (Exo) loaded with Que (Exo‐Que)	Rat	Intraperitoneal	Relieving the symptoms of AD by inhibiting phosphorylation of Tau and reducing the formation of insoluble neurofibrillary tangles (NFTs)	[250]
PD	mouse blood serum EVs loaded with Dopamine	Mice	Intravenous	Showing much better therapeutic efficacy in a PD model and lower systemic toxicity; Brain distribution of dopamine increased >15‐fold	[251]
PD	miR‐188‐3p‐modified mouse ADMSCs EVs	Mice	Intravenous	suppressed autophagy and pyroptosis, whereas increased proliferation via targeting cell division protein kinase 5 and NLRP3	[252]
PD	Mouse macrophage EVs loaded with catalase (exoCAT)	Mice	intranasal	Providing significant neuroprotective effects	[127a]
PD	RVG‐modified exosomes loaded with Catalase mRNA or α‐syn DNA aptamers Human HEK‐293T cells	Mice	subcutaneous/Intraperitoneal	Reducing the pathological α‐synuclein aggregates, neuroinflammation, and neuronal cell death; Improving motor impairments Attenuating and	[253]
PD	hucMSCs‐EVs	Rat	Intravenous	Reaching the substantia nigra through BBB, relieving asymmetric rotation, reducing substantia nigra dopaminergic neuron loss and apoptosis, and upregulating the level of dopamine in the striatum.	[254]
PD	RVG‐modified exosomes loaded with Curcumin, siSNCA or shRNA minicircles, or α‐syn siRNA from Mouse DCs	Mice	Intravenous	Clearing α‐synuclein aggregates and reducing reduced the loss of dopaminergic neurons and cytotoxicity	[255]

## 7 Summary and Outlook

COVID‐19 is not solely a lung disease but rather a systemic immune vascular disease involving multiple organs and tissues, particularly the nervous system.^[^ ^256^ ^]^ The incidence and severity of neurological complications of COVID‐19 are of increasing concern. As entities that transmit multi‐level information between the central and peripheral systems, EVs have established roles in the neuropathophysiology of SARS‐CoV‐2. Secretory vesicles serve as the primary mode of SARS‐CoV‐2 efflux, and EVs can assist in the transport of SARS‐CoV‐2 receptors. Moreover, the excessive production of proinflammatory and procoagulant EVs is an essential link in the central chain reaction of cytokine storm and coagulation disorders in COVID‐19. Notably, EVs have demonstrated the ability to freely access the BBB, suggesting their potential as a bioinformatic tool to predict the progression of COVID‐19‐related neurological complications or as a nanoscale targeted delivery system for specific therapeutic cargo to exert central therapeutic effects. However, several unanswered questions remain regarding the role of EVs in COVID‐19 neurological complications. First, it is crucial to acknowledge the long‐lasting neurological damage caused by COVID‐19 even after the epidemic ends, necessitating ongoing vigilance and comprehensive neurological examination and collection of pathological specimens. Second, there is a lack of pathological samples specifically used for EVs research, representing a favorable approach for understanding the connection and mechanisms between EVs and various brain tissue injuries. Third, the viral activity of EV‐carried viruses or viral components in brain tissue or CSF has yet to be determined, leaving uncertainty about whether viral replication or viral components alone are responsible for neuropathology. Moreover, while existing information focuses on the facilitation or causation of central damage by COVID‐19, there is a dearth of knowledge concerning the specific mechanisms by which EVs and their contents contribute to neuropathology, requiring meticulous research. Besides, research on EVs in the context of SARS‐CoV‐2 is associated with high risk due to the potential for EVs to carry the virus.Taken together, although circulating brain‐derived EVs have shown promising predictive and diagnostic potential regarding COVID‐19 neuropathy, studies in this area remain preliminary and limited in sample sizes. And the proposed use of EVs as a therapeutic delivery system for COVID‐19 neurological lesions raises numerous questions regarding cargo selection, safety enhancement, and brain targeting. It is undeniable that the field of EVs in the pathology, prediction, diagnosis, and treatment of COVID‐19‐related neurological complications requires further attention, effort, and extensive preclinical data and evaluation with larger patient cohorts before the clinical application can be considered.^[^ ^9b^ ^]^

In conclusion, although the COVID‐19 pandemic is essentially over, its impact on the organism may be long‐lasting. EVs and viruses are two intertwined entities, and much of the knowledge referred to in our review is generalized between various viruses and EVs, which prompts us not to stop focusing on and exploring the knowledge between EVs and viruses or virus‐associated neuropathology.

## Conflict of Interest

The authors declare no conflict of interests.

## Author Contributions

H.C., E.C., and Y.H. contributed equally to this work. C.C. and J.L. had the idea for the manuscript. H.Q.C., E.Y.C., and Y.H. performed the literature search, wrote the manuscript, and contributed equally to this review. L.N.W. and L.Y.D. collected data from previously published articles for Figure 1. C.C., J.L., T.Z., S.X.Y.‐L, and X.B.M. critically revised this manuscript. All authors reviewed, edited, and agreed on the content of the manuscript before submission.
